# Chart review of acute myocardial infarction at a district hospital in KwaZulu-Natal, South Africa

**DOI:** 10.4102/phcfm.v8i1.1012

**Published:** 2016-03-30

**Authors:** Roland Chetty, Andrew Ross

**Affiliations:** 1 Department of Family Medicine, Northdale Hospital, KwaZulu-Natal, South Africa; 2 Department of Family Medicine, University of KwaZulu-Natal, South Africa

## Abstract

**Background:**

Incidence and prevalence of non-communicable diseases, including ischaemic heart disease (IHD) and associated acute myocardial infarction (AMI), are increasing in South Africa. Local studies are needed as contextual factors, such as healthcare systems, gender and ethnicity, may affect presentation and management. In AMI, reviews on time between onset of chest pain and initiation of urgent treatment are useful, as delays in initiation of thrombolytic treatment significantly increase morbidity and mortality.

**Aim:**

The aim of the study was to determine the profile and management of patients admitted with ischaemic chest pain.

**Setting:**

The study was carried out in a busy urban-based district hospital in KwaZulu-Natal, South Africa. The population served is poor, and patients are mainly Indian with associated high risk of IHD.

**Methods:**

A chart review of all patients seen at the hospital with acute ischaemic chest pain between 01 March and 31 August 2014 was undertaken.

**Results:**

More male than female patients were admitted, with a wide variation in age. Most eligible patients received required thrombolytic intervention within an acceptable time period after arrival at hospital.

**Conclusion:**

Chest pain and AMI were a relatively common presentation at the study site, and urgent diagnosis and initiation of fibrinolytic therapy are essential. The encouraging door-to-needle time may have been influenced by the availability of specialist family physicians, trained as ‘expert generalists’ to provide appropriate care in a variety of settings and consultant support to junior staff. The role of the family physician and primary healthcare doctor in primary prevention are re-emphasised through the study findings.

## Introduction

South Africa is in the midst of a profound health transition, which is characterised by a quadruple burden of disease: communicable, non-communicable, perinatal and maternal and injury-related disorders.^1^ Historically, non-communicable diseases (NCDs) have been associated with relatively affluent countries; however, mortality from NCDs in developing world countries is significant. For example, in 2008 there were 28 million deaths because of NCDs in low-income countries, with 17 million deaths directly attributable to ischaemic heart disease (IHD).^2^

South African statistics indicate an annual mortality associated with IHD in the Indian population of 206.9 per 100 000 members of the population and of 162.7 per 100 000 in the white population. Mortality in the African/black population from IHD was 13.1/100 000.^3^ In South Africa, NCDs are increasing in incidence and prevalence in both rural and urban areas, most prominently amongst poor people living in urban settings, resulting in increasing pressure on acute and chronic healthcare services.^1^

Since 1994, the healthcare system in South Africa has been re-engineered and reprioritised, from specialist, tertiary care towards a primary healthcare (PHC) approach with emphasis on prevention, early detection and management of common conditions at local clinic or district hospital level.^4^ Family Physicians, working as ‘expert generalists’ in PHC facilities and district hospitals, strengthen PHC services by providing care to patients and consultant support to junior medical staff and nursing colleagues, build capacity through training and provide leadership in clinical governance.^5^ In addition, standard treatment guidelines have been developed which provide evidence-based guidelines for the management of patients who present to district hospitals and PHC facilities with common problems, including those who present with acute-onset chest pain.^6^

Management of patients diagnosed with ST-elevation myocardial infarction (STEMI) involves assessment and administration of reperfusion agents, with referral to a specialist centre when stabilised or not responding to reperfusion therapy. This has advantages in that cardiologists at secondary and tertiary centres can focus on the management of more complicated patients with IHD and patients with chest pain can be readily and speedily assessed at the district hospital, which contributes towards an accessible and affordable healthcare system. However, there are potential disadvantages: for example, doctors at district hospitals may be relatively inexperienced in evaluating acute-onset chest pain and diagnosing STEMI, and there may be resultant errors in diagnosis and management of these patients.

Acute myocardial infarction (AMI) mainly takes one of two forms: (1) STEMI and (2) non–ST-elevation myocardial infarction (NSTEMI). In STEMI, morbidity and mortality can be reduced by early interventions, such as fibrinolysis or percutaneous coronary intervention (PCI). There is evidence to show that early PCI is more advantageous in reducing mortality from reinfarction and reducing the need for a coronary artery bypass graft than fibrinolytic drug therapy.^7^ However, in South Africa, PCI is generally limited to tertiary hospitals, making fibrinolytic drug therapy the more accessible form of treatment for STEMI patients.

Urgent thrombolysis using fibrinolytic therapy is vital in STEMI to restore blood flow in an obstructed coronary artery. Such urgent intervention is a key factor for positive short- and long-term outcomes.^8^ Guidelines from the American Heart Association recommend a timeline of less than 30 min from arrival in the emergency department to fibrinolysis for STEMI, referred to as the ‘door-to-needle time’.^9^ Given the importance of minimising door-to-needle time, it is appropriate to review the management of AMI at the district level of care in a South African setting as survival is dependent upon urgent assessment and initiation of appropriate management.^8^ Local studies are needed as contextual factors, such as healthcare systems, gender, ethnicity and associated comorbidity, may affect presentation and management. Reviewing the profiles of patients with NCDs such as IHD is also important to guide local policy-makers in planning of services, particularly in a country such as South Africa where the burden of NCDs is growing and the morbidity and mortality associated with IHD can be influenced by multiple contextual factors and door-to-needle time within the re-engineered PHC system.

The study reviewed the incidence and management of patients who presented with AMI secondary to IHD at an urban-based district hospital in KwaZulu-Natal, South Africa.

## Research methods and design

The design was retrospective and descriptive. The study site was a 366-bed urban-based district hospital in KwaZulu-Natal. The hospital serves a large population, and the majority of patients are classified as Indian. In South Africa, any Indian population can be expected to have a relatively high prevalence of NCDs, including hypertension, diabetes and IHD.^10^ The district hospital has a casualty department that is staffed by junior medical officers with senior support provided by specialist family physicians in the Department of Family Medicine.

Patients presenting with acute-onset chest pain are reviewed in casualty by the medical officer on call, and those with suspected AMI are discussed with family physicians based at the hospital, who in turn discuss patients with the cardiology department at a regional referral hospital; electrocardiographs (ECGs) are faxed to the regional hospital. Thrombolytic therapy can thus be provided at this district hospital under the supervision of a family physician and cardiologist, with the aim of ensuring accurate diagnosis and minimising door-to-needle time. On completion of thrombolytic therapy, patients are then transferred to the coronary care unit at the regional hospital. Patients who present with NSTEMI, those with STEMI who present after the ‘window of opportunity’ for thrombolysis and patients with contraindications for thrombolysis are generally admitted to the high-care unit at the study site.

Data collection involved hospital chart review of all consecutive patients admitted with acute-onset chest pain over a 6-month period (01 March to 31 August 2014). In this setting, hospital charts are paper based and stored alphabetically by surname. The names of patients with acute-onset chest pain and AMI (STEMI or NSTEMI) were identified via casualty records, and charts were drawn by the researcher from the hospital record department.

Data were gathered using a standardized data collection sheet, which considered demographic and clinical details, including risk factors for AMI. Only charts with all the required information were considered for inclusion in the study. The data collection sheet was validated for content using a pilot of 10 hospital charts. Criteria for a chart to be considered for inclusion included (1) a diagnosis of STEMI, which was based on a history of acute-onset chest pain, an ECG showing ST-elevation and an elevated troponin I level and (2) a diagnosis of NSTEMI, which included a history of acute chest pain, with or without ischaemic changes on ECG (ST depression, T-wave inversions) and an elevated troponin I level. Data were entered into the SPSS programme and analysed descriptively.

## Ethical considerations

Ethical approval for this study was given by the Research Ethics Committee of the University of KwaZulu-Natal (BE 445/2014), and relevant permission was obtained from the Department of Health and managers at the study site.

## Results

A total of 149 patients presented to the casualty department with acute-onset chest pain and AMI during the study period, giving an average of 25 patients per month. One hundred and twenty-two (122/149; 82%) hospital charts met the criteria for inclusion in the study. There was incomplete information in 21 charts, and 6 patients did not meet the inclusion criteria as a diagnosis of AMI could not be confirmed.

The mean age of patients was 61 years (range, 42–84 years) and just over two-thirds (79; 65%) were male patients. The majority were classified as Indian (73; 60%), whilst 30 (25%) were white people and 19 (15%) were black people. There were slightly more patients diagnosed with NSTEMI (67; 55%) than STEMI (55; 45%). Demographic details are summarised in Table 1.

**TABLE 1:** Demographic details of patients admitted with acute myocardial infarction.

Variable	ST-elevation myocardial infarction	Non-ST-elevation myocardial infarction
**Age**		
40–49	5	7
50–59	12	17
60–69	29	32
70–79	5	6
> 80 years	4	5
Total *n* (%)	55 (45)	67 (55)
**Gender**		
Male patients	34	45
Female patients	21	22
**Race**		
White people *n* (%)	13 (24)	17 (25)
Indian *n* (%)	31 (56)	42 (63)
Black people *n* (%)	11 (20)	8 (12)
Mixed race *n* (%)	0	0

Hospital chart review indicated that most patients smoked (85; 70%), and the prevalence of diabetes was high (73; 60%). Just under half had hypertension and a quarter had recorded dyslipidaemia. One-third had a known history of IHD and half had a family history of IHD. These risk factors are summarised in Table 2.

**TABLE 2:** Risk factors for acute myocardial infarction.

Variable	Smoking *n* (%)	Diabetes *n* (%)	Hypertension *n* (%)	Dyslipidaemia *n* (%)	Known ischaemic heart disease *n* (%)	Family history of ischaemic heart disease *n* (%)
ST-elevation myocardial infarction	37	32	26	14	19	33
Non-ST-elevation myocardial infarction	48	41	29	17	12	28

**Total**	**85 (70)**	**73 (60)**	**55 (45)**	**31 (25)**	**31 (25)**	**61 (50)**

Most patients with STEMI (43; 78%) received thrombolysis at the study site. Reasons for not receiving thrombolysis included late presentation after onset of chest pain (7; 13%) and contraindications to thrombolysis (4; 7%). The mortality rate in patients with STEMI was 1/55. These data are presented in Table 3. The average door-to-needle time was 43 min (range, 25–60 min).

**TABLE 3:** Outcome summary for patients with ST-elevation myocardial infarction (*n* = 55).

Outcome	Number (%)	Reason
Thrombolysed	43 (78)	-
Not thrombolysed	12 (22)	Late presentation 7 Contraindications 4 Died – cardiogenic shock 1 (2%)

Most patients with NSTEMI were referred to the cardiac unit at the regional hospital (43; 64%) and a quarter were admitted to the high-care unit at the study site (20; 22%). A total of 92 (75%) patients with AMI were transferred to the regional cardiac unit during the study period (Table 4).

**TABLE 4:** Outcomes of patients with non–ST-elevation myocardial infarction (*n* = 67).

Outcome	Number (%)
Transferred to cardiologist	43 (64)
Admitted to high-care at the study site	20 (22)
Refused admission	4
Refused admission	
**Total**	**67**

## Discussion

Policy planners at this study site should expect the admission of approximately 25 patients with AMI monthly. These patients require urgent and accurate diagnosis and intervention, as the time to treatment is a pivotal parameter in reperfusion, and a prolonged interval between onset of symptoms and thrombolysis in STEMI can result in significant morbidity and mortality.^9^ It was encouraging that the door-to-needle time was on average 43 min (range, 25–60 min). This time is set against the challenges associated with working in a busy casualty department with predominately junior staff in a South African setting, where consultation with a family physician and an offsite specialist was required. This door-to-needle time is close to the 30 min recommended by the American Heart Association.

Other studies from Cape Town have reported a door-to-needle time of 54 min; initiation of thrombolysis at these sites was delayed by factors including delays in junior doctors being able to access advice from more senior colleagues, and difficulties in reading ECGs.^11^ The Cape Town study recommended that a key modifiable factor contributing to prolonged door-to-needle times was the need for senior review or for advice on ECG interpretation; this contributed to almost half of the documented delays in thrombolysis.^11^

The key finding in the current study of a positive door-to-needle time of 43 min was considered to be related to the availability of specialist family physicians who are well trained in assessing chest pain and in communicating with colleagues. Other studies support the positive influence of well-trained personnel in casualty departments, who can liaise with offsite specialists in making decisions around ongoing care of patients with AMI.^12^

It was also encouraging that the majority of hospital charts contained the required basic information for inclusion into the study. However, it is of concern that 21/149 (13.5%) did not contain basic information on demography and risk factors. A need for good record-keeping is set against the high turnover of staff and the relatively junior status of staff in the casualty department of this district hospital. Limitations of paper-based charts are discussed in South African literature, and studies advocate for the use of electronic records, which may lead to more complete information becoming widely available on disorders including AMI.^13^

There were more male than female patients admitted with AMI, and this finding is supported in other South African literature on gender and AMI.^11^ The profile of patients seen was not unexpected as the hospital provides services for a large Indian population who have a high incidence of diabetes, hypertension and hypercholesterolaemia.^9^ The male preponderance is also in keeping with international studies, which have demonstrated that male gender is an independent risk factor for IHD and AMI.^1^ A more in-depth study of gender and AMI may prove useful as literature indicates that female gender in STEMI is associated with a lower chance of receiving urgent thrombolytic intervention.^14^ Female gender may negatively bias the opportunity for acute intervention because the ECG changes in female patients with STEMI can present very subtly with lower total ST-segment elevation than in male patients.^15^

It is, however, important to note the 19 black patients who presented with IHD, which highlights the growing number of black African patients presenting with IHD because of urbanisation and a change in dietary patterns amongst the African population, and the importance of considering IHD in all patients presenting with acute chest pain.^16^

There was a wide range of ages of patients with AMI, and the relatively young presentation (42 years) may reflect the high risk of IHD in a South African Indian population. The average age of 61.4 years (range, 42–84 years) is similar to the age of presentation of AMI in Western populations, as documented in the Courage Trial.^17^

The high percentage of patients who smoked, were diabetic and had a family history of IHD is consistent with other studies, which have demonstrated an association between these risk factors and AMI.^18,19,20^ The emphasis on PHC in South Africa highlights the importance of all doctors, particularly those working in PHC settings, actively engaging with their patients at every opportunity to help them stop smoking. Even brief interventions based on the 5 As (ask, alert, assess, assist and arrange), which could be incorporated into routine practice, have been shown to be effective in helping patients to stop smoking.^21^ In addition, it is important that doctors use appropriate guidelines and treat patients with diabetes, hypertension and hypercholesterolaemia. The review re-emphasises that PHC doctors should identify patients at risk for developing IHD, ensure optimal control of any comorbid conditions and assess regularly.

## Limitations

This study was carried out at one study site and findings may be strengthened by expanding the study to other sites. The data collection method was retrospective and relied on the accuracy of data recorded; there is a possibility that patients who were not diagnosed correctly were excluded from the study. It may be useful to follow a cohort of patients with chest pain from admission to diagnosis to more fully understand and document the diagnosis and management of chest pain at the study site.

## Conclusion

Chest pain and AMI were a relatively common presentation at the study site, and urgent diagnosis and initiation of fibrinolytic therapy where appropriate are essential. The encouraging door-to-needle time may have been influenced by the availability of specialist family physicians, trained as ‘expert generalists’ to provide appropriate care in a variety of settings and consultant support to junior staff.^5^ The roles of the family physician and PHC doctor in primary prevention are re-emphasised through the study findings.
